# Six-month healing success rates after endodontic treatment using the novel GentleWave™ System: The pure prospective multi-center clinical study

**DOI:** 10.4317/jced.52779

**Published:** 2016-07-01

**Authors:** Asgeir Sigurdsson, Khang T. Le, Stacey M. Woo, Shahriar A. Rassoulian, Kimberly McLachlan, Farah Abbassi, Randy W. Garland

**Affiliations:** 1 DDS, MS. Department of Endodontics, New York University College of Dentistry, New York; 2 DDS. Private Practices, Santa Ana, California; 3 DDS, PhD. Private Practices, Whittier, California; 4 DMD. Private Practices, Aliso Viejo, California; 5 DMD, MSEd, MBA. Private Practices, Encinitas, California; 6 DMD, MSD. Private Practices, Santa Ana, California; 7 DDS. Private Practices, Encinitas, California

## Abstract

**Background:**

This prospective multi-center (PURE) clinical study evaluated healing rates for molars after root canal treatment employing the GentleWave® System (Sonendo, Inc., Laguna Hills, CA).

**Material and Methods:**

Eighty-nine patients met the inclusion criteria and consented for this clinical study after referral for a root canal treatment. All enrolled patients were treated with the GentleWave System. Five endodontists performed the clinical procedures and follow-up evaluations. Pre-operative, intra-operative, and post-operative data were collected from the consented patients. Each patient was evaluated for clinical signs and symptoms. Two trained, blinded, and independent evaluators scored the subject tooth radiographs for apical periodontitis using the periapical index (PAI). The teeth classified as healing or healed were considered as a success and composed of a cumulative success rate of healing. Statistical analysis was performed by using the Fisher’s exact test, Pearson correlation, and multivariate logistic regression analyses of the pre-operative prognostic factors at 0.05 significance level.

**Results:**

Seventy-seven patients were evaluated at six months with a follow-up rate of 86.5%. The cumulative success rate of healing was 97.4%. Eleven prognostic factors were identified using bivariate analyses. Using logistic analyses, the two prognostic significant variables that were directly correlated to healing were the pre-operative presence of periapical index (*p* value=0.016), and single treatment visits (*p* value=0.024).

**Conclusions:**

In this six-month PURE clinical study, the cumulative success rate of healing was 97.4% when patients were treated with the GentleWave® System.

** Key words:**Healing rate, root canal treatment, molar, GentleWave™, Sonendo®, Multisonic Ultracleaning™ .

## Introduction

Endodontic treatment aims to remove vital and/or necrotic tissue, bacteria and bacterial irritants from the root canal system, thereby promotes healing of the periapical area (1-2). Hence, complete root canal cleaning and disinfection is essential to achieve healing of periradicular tissue and successful endodontic treatment (3).

Many etiological factors affect the outcome of endodontic treatment (4). It is well accepted that current cleaning and shaping procedures cannot reach all the intricacies of the root canal system (5). As such, chemo-mechanical preparation and instrumentation do not always completely eradicate the tissue or microbiota present in the anatomical complexities of the root canal system (6).

Different irrigation techniques and devices have been developed to improve the cleaning of the root canal system, including ultra-sonic irrigation, negative pressure irrigation, sonic irrigation, photo-induced photo-acoustic streaming (PIPS), and laser technologies. However, the safety, efficacy, and/or reliability of all these techniques have been questioned in many studies (7-13). The positive pressure induced by some of these techniques may result in irrigant extrusion to the peri-apex, which may lead to severe patient trauma and post-operative pain (7-10). Further, tissue debris and biofilm cleaning of even contemporary techniques is often insufficient to provide an environment conducive for long term success (2,9-11). Furthermore, most of these techniques require increased dentin removal from the roots to facilitate the penetration of irrigants into the root canal system, which may weaken the remaining tooth and thereby also negatively affect long-term healing rates (12,13).

The GentleWave® System (Sonendo, Inc., Laguna Hills, CA), which consists of a console and a treatment instrument should be capitalized, has been developed as a novel approach to clean and disinfect the root canal system (14-17). Haapasalo *et al.* (2014) demonstrated that the tissue dissolution efficacy of the GentleWave® System is at least eight times greater than that of conventional irrigation systems, ultrasonic irrigation, and EndoVac (14). Ma *et al.* (2015) performed micro-CT analysis and compared the cleaning efficiency of the GentleWave® System with passive ultrasonic system and conventional needle irrigation configuration. The authors showed cleaning of the entire root canal system including the apical-third regions (15). The GentleWave System was the only technique that removed all the calcium hydroxide even in the apical thirds. However, these studies were performed *in-vitro* using extracted teeth. While *in-vitro* studies have demonstrated excellent results by the GentleWave® System with regards to canal cleanliness and safety, it is ultimately the *in-vivo* clinical studies that are needed for higher level evidence of the performance and benefits of any endodontic treatment strategy or device. The current study is the first clinical research that reports the healing rates observed by five independent endodontists utilizing the GentleWave® System.

## Material and Methods

-Study cohort

The inception cohort comprised of eighty-nine patients who were referred for an endodontic treatment. The study protocol for the multi-center, prospective, non-significant risk clinical study was approved by an Institutional Review Board (Aspire Llc) and the study was carried out in accordance with the Declaration of Helsinki. The clinical study evaluated the healing rates of endodontic treatments performed using the GentleWave® System. The purpose of the study was explained to the patients and written informed consents were obtained. All the subjects adhered to previously defined inclusion and exclusion criteria stated in  Table 1. After initiation of the study, the subjects were given the opportunity to withdraw. A total of 89 teeth, one tooth per patient, were treated for the clinical study.

**Table 1:**
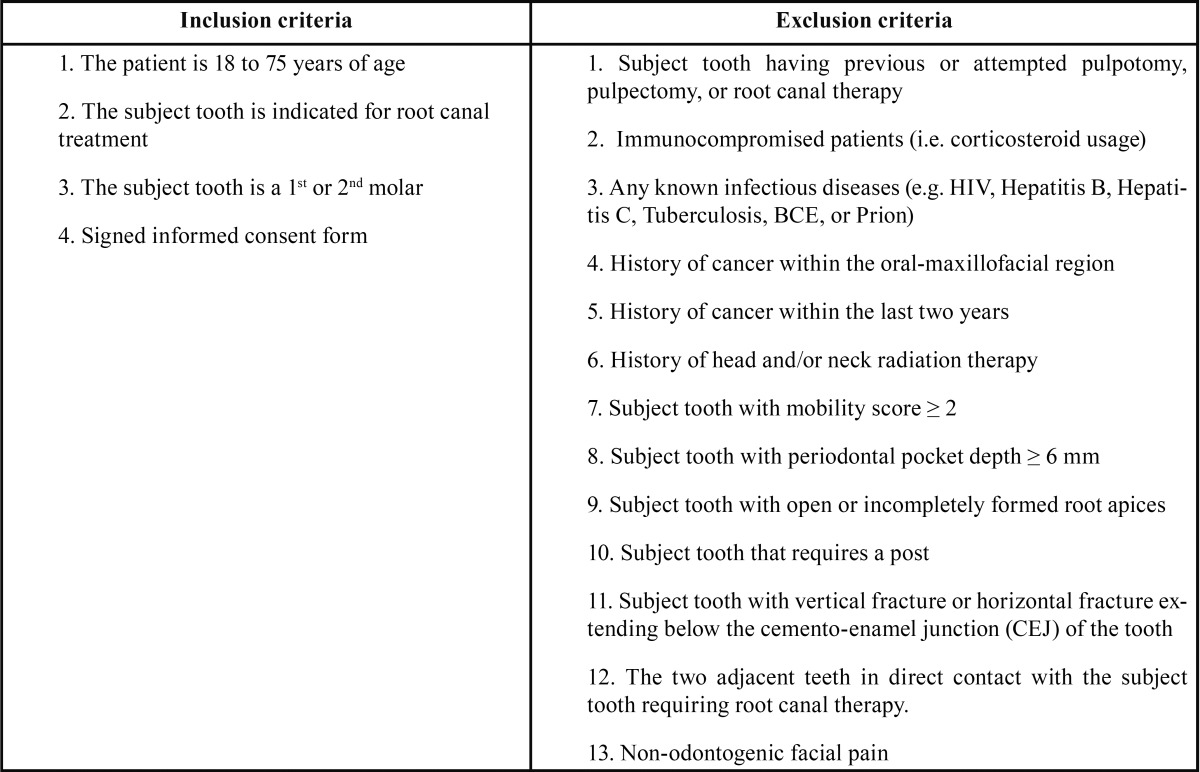
The PURE clinical study inclusion and exclusion criteria.

-Intervention

Five endodontists participated as investigators in the multi-center, prospective, non significant risk clinical study to assess the long-term performance of the Sonendo® endotherapy system (PURE). The investigators were trained for using the GentleWave System and performed a standardized treatment procedure at their independent clinical sites. Using standard coded data sheets, the collected redacted clinical and radiographic data pertained to each treated tooth before (pre-operative), during (intra-operative), and six-months after (post-operative) the initial treatment. The data was directly transferred to a database.

-Pre-operative data collection 

Prior to treatment, the patients were clinically examined and radiographs were taken. Pulp and periradicular diagnosis was completed and regarded.

-Treatment procedure

**Figure 1:**
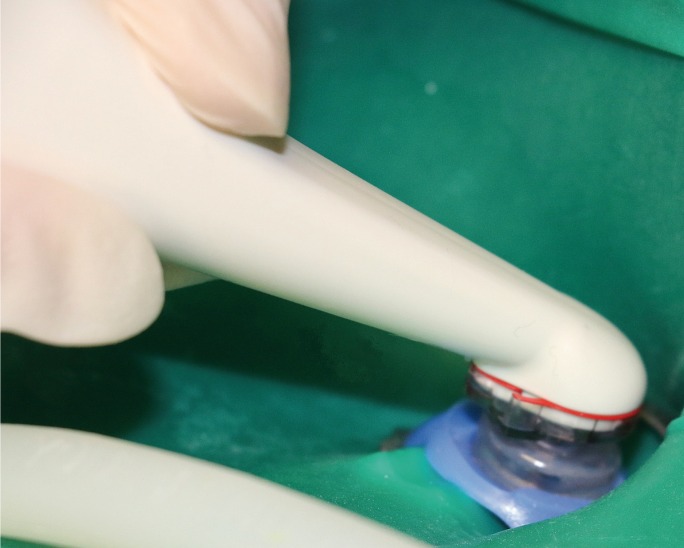
The handpiece of the GentleWave System placed on the subject tooth. The treatment instrument does not enter the tooth but sits on a sealed platform. The tip of the handpiece enters the pulp chamber of the tooth.

The patient was anesthetized per standard techniques, the type of injection being at the discretion of the endodontist. The tooth was isolated with dental dam. Caries and existing restoration were removed. Missing tooth structures were built-up and a conservative straight-line access was performed. Patency was confirmed with #10 and #15 K type hand files (MANI K files, Utsunomiya, Japan) and the working length (defined as distance to the apical constriction of approximately 0.5-1 mm from the radiographic apex) was achieved using electronic apex locator and confirmed with radiographs. Teeth were instrumented with a standardized minimal instrumentation protocol that included the use of hand files up to size ISO #20 and Protaper file F1 (Dentsply, Tulsa Dental Specialties, Tulsa, OK) regardless of the initial canal size. The GentleWave treatment instrument should be capitalized was then placed on the endodontic access opening of the molars as shown in figure 1. The treatment consisted of up to 3% sodium hypochlorite (NaOCl, Clorox, Oakland, CA), a distilled water rinse, up to 8% ethylenediaminetetraacetic acid (EDTA, Vista, Racine, WI), and a final distilled water rinse 30 seconds, 8% ethylenediaminetetraacetic acid (EDTA, Vista, Racine, WI) for 2 minutes, and distilled water for 15 seconds (17). Canals were subsequently dried with absorbent paper points. The dried canals were obturated using warm vertical technique with gutta percha and AH Plus® sealer (Dentsply, Tulsa Dental Specialties, Tulsa, OK). The pulp chamber floor was sealed with bonded composite and the patients went to the referring general dentist for final post treatment restoration.

-Intra-operative data collection

During the treatment, the final apical diameter, calcification, type of obturation, the root filling length, sealer extrusion if any, and coronal seal were documented.

-Post-operative data collection

Post-treatment symptoms were assessed two days after the treatment using a visual analog scale (VAS; 0 and 10) to rank the level of experienced pain (18). Each investigator completed a follow-up assessment every three months for patients enrolled at their respective clinical site. Assessments were standardized and included both clinical and radiographic examinations. The clinical examination involved an update on the medical and dental history, intra oral evaluation which included periodontal pocket depth measurements, mobility testing, presence and extent of swelling and soft tissue lesion, and assessment of percussion and palpation.

-Outcome measures and criteria

Teeth were assessed for healing utilizing a composite endpoint which included both clinical and radiographic components. Clinical signs and symptoms as discussed previously were utilized for assessing the clinical component. Periapical index scoring (PAI) was utilized to assess the tooth using a periapical radiograph. The scores ranged from 1 (for normal periradicular tissue) to 5 (severe periodontitis with exacerbating features) (19).

Based on clinical signs/symptoms and PAI scores, teeth were classified as healed, healing, or diseased (19,20).

In summary, the diagnosed teeth were classified as follows:

(a) Healed – clinical normalcy other than tenderness to percussion accompanied by radiographic PAI scores of 1 or 2.

(b) Healing – clinical normalcy other than tenderness to percussion accompanied by reduction in the size of periradicular lesion or reduction in PAI score.

(c) Diseased – presence of clinical signs and symptoms accompanied by radiographic PAI score of 3 or higher or increase in the size of periradicular lesion or increase in PAI score.

The teeth classified as healing or healed were considered as a success. The combined success of these cases was termed as healing rate.

-Calibration of evaluators 

The radiographs were blindly evaluated by two experienced endodontists. The images were coded and provided to the evaluators after being randomized between different patients. Before evaluating the images, the two examiners evaluated a series of radiographs independent of the study sample that represented a wide range of periapical lesions to account for inter-observer reliability (19). The Cohen’s kappa score was calculated. The exercise was independently performed three times to increase the calibration. In general, each visible root on the radiographs was assigned a PAI score. The highest PAI score for all the roots for a given tooth was considered as the PAI score of the tooth. This PAI score was considered for further statistical evaluation.

-Evaluating radiographs 

The two evaluators independently scored the radiographs. After the independent scoring sessions, the examiners reached an agreement on the PAI scores if the scores of their independent evaluations differed. The consensus scores for all the radiograph images were considered as the true score and were used for statistical analysis.

-Statistical analysis 

All the tests were performed as two-tailed with SPSS 15.0 (SPSS Inc., Chicago, IL) at 5% level of significance. When analyzing, the event of interest was the success of healing of the tooth. A total of 34 variables were investigated. A univariate and bivariate analyses with percentage of frequencies and *p*-values was generated to characterize the study cohort. The bivariate analysis included outcome associations with pre-operative, intra-operative, and post-operative variables (Fisher exact test) to identify variables of interest. Spearson coefficients were calculated to determine any correlation between these variables to categorize potential outcome predictors. Finally, a multivariate analysis using logistic regression models was used to detect the significant outcome predictors. The odds ratio (OR) and confidence intervals (CI) were calculated.

## Results

-Examination reliability

The achieved Cohen’s kappa score for intra-observer agreement between the independent reviewers was 0.73- 0.75, indicating a good to very good agreement (19).

-Recall and healing 

Eighty-nine patients met the inclusion criteria and consented to participate in the clinical study. Of the 89 patients, 43.8% were male whereas 56.2% were female. 5.6% had a history of diabetes whereas 12.1% had a history of tobacco use. Oral hygiene of the study cohort was good (67.4%) or fair (32.6%).

The successful recall of 77 of 89 teeth of the available patients represented an 86.5% recall rate. Of the 77 teeth, 60 teeth (77.9%) were healed, 15 teeth (19.5%) were being healed, and two teeth (2.6%) were diseased. Overall, 75 of 77 teeth (97.4%) were being healed six months after the GentleWave treatments. These results are summarized in  Table 2,  Table 2 continue.

**Table 2:**
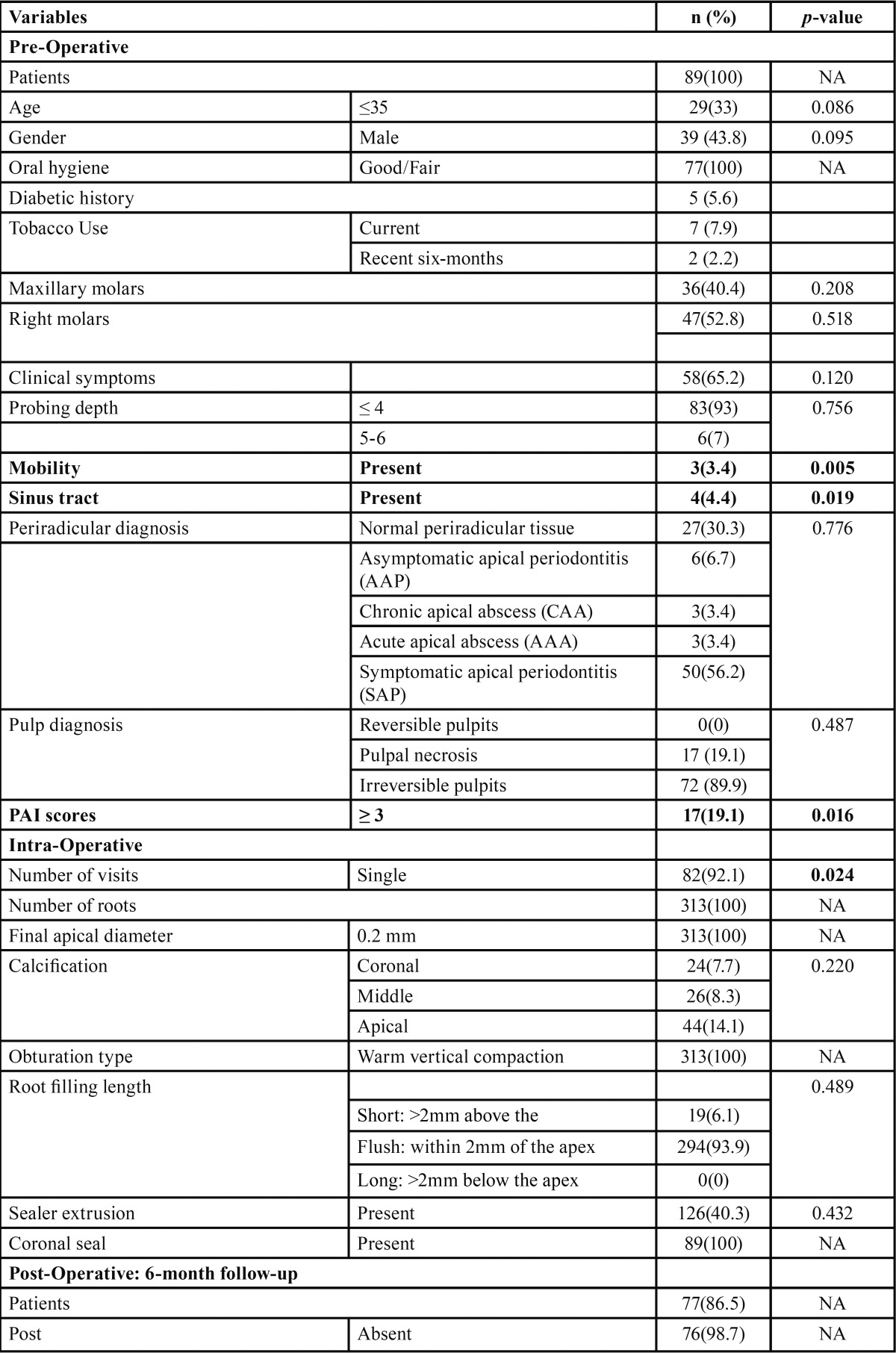
Bivariate analyses – unadjusted effects of pre-operative, intra-operative, and post-operative tooth factors compared with the event of healing.

**Table 2 continue:**
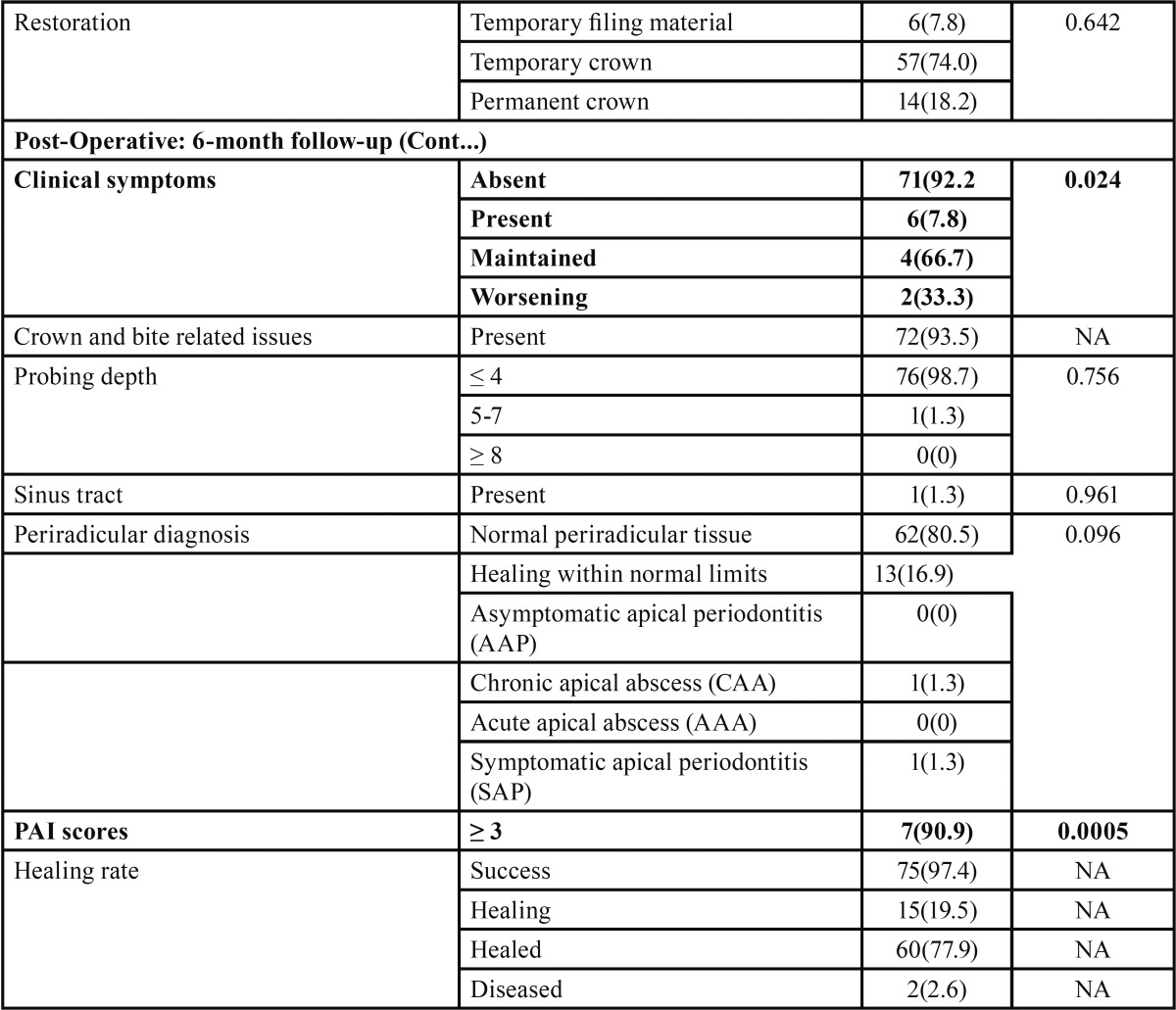
Bivariate analyses – unadjusted effects of pre-operative, intra-operative, and post-operative tooth factors compared with the event of healing.

-Identifying predictor factors

 Table 2 also provides an overview of the pre-operative, intra-operative, and post-operative factors.

Pre-operative factors: None of the pre-operative factors had a significant difference when compared to healing. The p values for gender, age, and oral hygiene were 0.86, 0.095, and 0.33, respectively. The following factors were also analyzed: periradicular diagnosis (*p*-value=0.787), pulp diagnosis (*p* value=0.487); PAI score (*p*-value=0.573), pocket depth (*p*-value=0.560), pre-operative symptoms (*p* value =0.258), maxillary versus mandibular molars (*p*-value =0.207) and right versus left molars (*p* value=0.120).

Inter-operative factors: A significant difference was observed (*p*-value=0.024) for the intra-operative factor related to single versus two-day endodontic treatment; the success rate of healing was correlated to single-visit treatments. Calcification (*p* value=0.221), sealer extrusion (*p*-value=0.998) and root canal filling length (*p*-value=0.507) demonstrated no significant difference in regards to healing.

Post-operative factors: At the six-month follow-up data was collected similar to that at the pre operative visit. Post-operative clinical symptoms (*p*-value=0.024) and post-operative PAI scores (*p*-value=0.0005) were significantly different. Periradicular diagnosis (*p*-value=0.096), pocket depth (*p*-value=0.756), and type of restoration (*p*-value=0.642) showed no significant difference.

Further, as shown in  Table 3, the Pearson correlations showed that the post-operative PAI score (r= 0.516; very significant), post-operative clinical symptoms, and number of visits (r=0.257; significant) directly correlated with healing. More specifically, favorable post-operative clinical symptoms and low PAI scores were observed with healing. Further, single-visit treatments were associated with healing. These three intra- and post-operative predictors were used for predicting the healing of periradicular lesions.

**Table 3:**
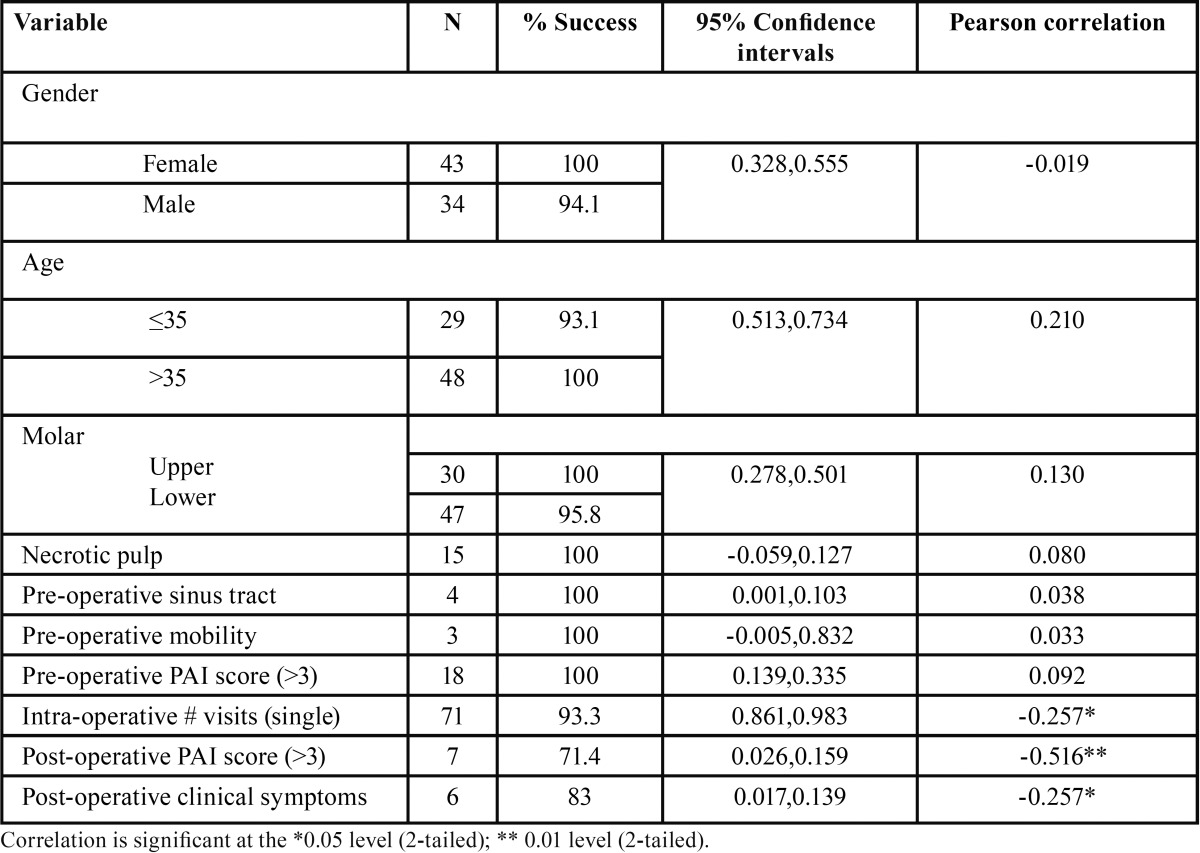
Bivariate analysis – confidence intervals and Pearson correlation of selected unweighted variables associated with the outcome (n=77).

Logistic regression model of the pooled sample ( Table 4) revealed that the healing rates are associated with the two pre- and intra-operative predictors: pre-operative PAI (OR=2.475; CI:0.434,14.127) and single visits (OR=37.747; CI:4.147,343.581). The Hosmer and Lemeshow test demonstrated that the predictive model ( Table 4) is a good fit.

**Table 4:**
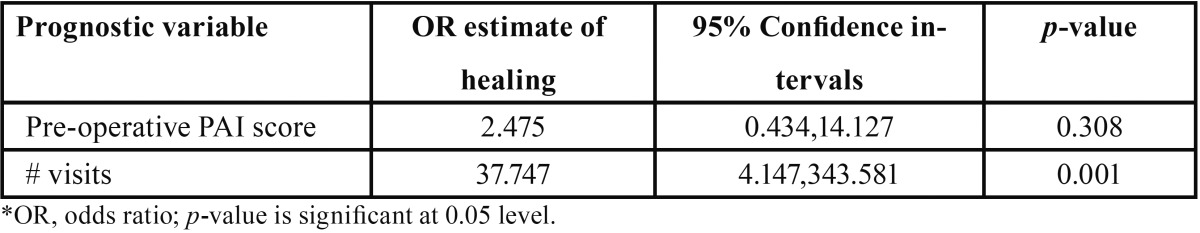
Logistic regression model identifying significant predictors of success after initial root canal treatment (n=77) with pre-operative periapical index and number of visits as prognostic variables.

## Discussion

The goal of endodontic treatment is to return the involved teeth to the best feasible state of health and function as soon as possible without surgical intervention (20). Previous clinical studies performed with different endodontic technologies show various healing rates at six-month follow-ups (11,20-23). Friedman *et al.* (1995) showed six month healing rates of 74% and 66% respectively, when the obturation material was varied (21). Murphy *et al.* (1991) retrospectively quantified the rate of healing of periapical radiolucencies after nonsurgical endodontic therapy as 17.6% (22). When teeth were treated with Er,Cr:YSGR laser and compared with those treated with conventional syringe-needle irrigation, the six month healing rates were 59% and 67% respectively (11). In another multicenter prospective study, Asgary *et al.* (2013) presented the healing rates to be 77.2% when vital pulp therapy with calcium-enriched mixture cement was utilized (23). Based on a random-effects meta-analysis, Ng *et al.* (2007) revealed the weighted-pooled success rates of healing to be 29.6% for a six month time period (2). Further, it has been suggested that at least a one year time period is needed to visualize substantial healing (4,19-20,24). Even when surgical intervention was employed and success rates were analyzed after a prolonged time, Chong *et al.* (2003) reported a success rate of 92% in over three years whereas Barone *et al.* (2010) reported a success rate of 74% after 10 years (24-25).

In contrast, the present study had a cumulative success rate of healing of 97.4% (75 patients) within six months. The exhibited faster healing rate in this clinical study may be associated with increased eradication of tissue debris, biofilm, and bacteria from the root canal system, as previously shown *in-vitro* (17). Histological analyses showed 97.2% of tissue debris in apical and middle region of mesial roots of mandibular molars, including isthmi, was removed after treatment with the GentleWave System.

Unfortunately, post-operative pain is common after endodontic treatment (18,26-28). Post treatment pain can be caused by extrusion of root canal microbes into the periapical area, over instrumentation, or by extrusion of the irrigation solution, NaOCl in particular. The incidence of post-operative pain was reported to range from 3% to 58% (26). According to Ng *et al.* (2007), 12% of the patients experience severe pain within two days after treatment (27). In the present study, no patients experienced severe pain (visual analog scale score≥9) while only 3% of the patients experienced moderate pain (visual analog scale score=7-8) within two days after the initial treatment. Conducive to this finding, the present study shows a three fold decrease in the patients that experienced pain two days after the initial treatment when compared with pre-operative scores. These results are coherent with the study performed by Gondim *et al.* (2010), where the authors show that the negative pressure system resulted in significantly less post-operative pain (28). Interestingly, Charara *et al.* (2015) compared the GentleWave System to EndoVac and showed that both these negative pressure systems led to zero extrusion to the periapical space *in-vitro* (16).

In recent years, single-visit appointment regimens have reported numerous advantages including better patient acceptance, reduction of the inter-appointment infection risks, saving time, and cost (9,29). However, significant post-operative pain has been reported after single-visit root canal treatments and for teeth having necrotic pulp (9,29). Xaio *et al.* (2010) compared the healing rates of one-visit appointment with two-visit appointments and concluded that the healing rates were 68.4% and 64.5% respectively (29). Beus *et al.* (2012) demonstrated the prevalence of bacteria remaining in the root canal system when teeth were treated with single-visit regimens (9).

On the contrary, in the present study using the GentleWave System, post-operative pain was not correlated with either single-visit appointment or with necrotic teeth. Moreover, 72 patients showed success (98.6%) when treated with single-visit endodontics whereas 15 patients showed success (100%) when the teeth were necrotic.

A fundamental factor that improves prognosis is the preservation of dentin structure in its native form (30). It is noteworthy that the present study utilized minimal endodontics by employing methods that minimally remove dentin structure while accessing the teeth and shaping the root canals. Previous studies showed that even when molars were shaped to #15/.04 *in-vitro* when using the GentleWave System, statistically significant clean root canal system was observed (14-17). However, the present clinical study utilized shaping to #20/.07 in order to facilitate standard obturation techniques. As shown in figure 1, the treatment instrument of the GentleWave System is placed in the pulp chamber of the molars. Since the treatment instrument should be capitalized does not have to enter the roots, the GentleWave System reduces the need for shaping of the roots using large instrumentation, hence practicing minimal endodontic technique with dentinal conservation. Details of the GentleWave technology are described elsewhere (16,17). Briefly, the technology employs a strong hydrodynamic cavitation cloud and generates a broad spectrum of sound waves that travel through the degassed treatment fluid and propagates throughout the entire root canal system.

The GentleWave System allows minimal instrumentation, cleans the root canal system thoroughly, and produces negative pressure in the root canal system (14-17). Therefore, the rare occurrence of post-operative symptoms in the present study after the tooth is treated with the GentleWave System, is not surprising.

In conclusion, root canal treatment utilizing the GentleWave System demonstrated a cumulative success rate for healing of 97.4% within six months of the initial treatment. Long term follow-ups can improve the statistical power and enable further investigation into prognostic factors for tooth healing following the root canal treatment. Additional *in vivo* studies are also needed to compare the healing rates acquired by the GentleWave System to those obtained with other conventional and contemporary endodontic techniques.
